# Bidirectional association between COPD and AF: a systematic review and meta-analysis

**DOI:** 10.3389/fcvm.2026.1840570

**Published:** 2026-06-19

**Authors:** Yunmeng Wang, Chaowei Ding, Yawei Liu, Jingchao Lu

**Affiliations:** 1 Department of Cardiovascular Medicine, The Second Hospital of Hebei Medical University, Shijiazhuang, Hebei Province, China; 2 Department of Respiratory and Critical Care Medicine, Xiamen Humanity Hospital Fujian Medical University, Xiamen, Fujian, China

**Keywords:** atrial fibrillation, bidirectional association, chronic obstructive pulmonary disease, meta-analysis, public health, systematic review

## Abstract

**Objective:**

This study aims to comprehensively evaluate the bidirectional epidemiological association between chronic obstructive pulmonary disease (COPD) and atrial fibrillation (AF), as well as their combined impact on prognosis, providing evidence to inform the clinical management of comorbidities.

**Methods:**

In accordance with PRISMA guidelines, a systematic literature search was conducted in PubMed, EMBASE, and the Cochrane Library from inception through June 2025, focusing on cohort and case-control studies. Study quality was assessed using the Newcastle-Ottawa Scale, and odds ratios (OR) were pooled using either random-effects or fixed-effects models to quantify the bidirectional risk between COPD and AF and their prognostic effects.

**Results:**

A total of 48 unique publications were included. The findings showed that AF was associated with a higher risk of COPD (OR = 1.56, 95% CI 1.06–2.29), and COPD was similarly associated with a higher risk of AF (OR = 1.72, 95% CI 1.42–2.09). Additionally, AF in COPD patients was associated with increased short-term (OR = 1.76) and long-term (OR = 1.27) mortality risks, while COPD in AF patients was also associated with increased mortality risks (short-term OR = 1.54, long-term OR = 1.68).

**Conclusion:**

COPD and AF showed a bidirectional observational association, and their coexistence was associated with increased mortality risk. Given the observational nature of the included studies and substantial heterogeneity, these findings should be interpreted as associations rather than causal effects. These findings highlight the need for integrated and individualized management strategies in patients with comorbid COPD and AF.

**Systematic Review Registration:**

https://www.crd.york.ac.uk/PROSPERO/view/CRD420251069785

## 1 Introduction

Atrial fibrillation (AF) is the most prevalent arrhythmia, holding a significant role in the global cardiovascular disease landscape (1). Its defining characteristic is the replacement of regular and coordinated electrical activity in the atria with rapid, irregular fibrillation, leading to cardiac rhythm disturbances, loss of atrial contraction efficiency, and compromised ventricular ejection function (2). Patients with AF frequently experience symptoms such as palpitations, chest tightness, dizziness, and fatigue, severely impacting their daily quality of life. Furthermore, AF substantially raises the risk of stroke, heart failure, and all-cause mortality (3). Epidemiological studies have indicated a concerning rise in the global prevalence of AF, closely linked to the aging population, making it a major public health concern for the elderly (4, 5).

Chronic obstructive pulmonary disease (COPD) is a chronic respiratory condition characterized by high incidence, disability, and mortality rates, representing a significant global health challenge (6). Its hallmark feature is persistent airflow limitation, commonly associated with an enhanced chronic inflammatory response in the airways and alveoli to harmful particles or gases (7). In recent years, accumulating evidence has highlighted the complex interaction between AF and COPD (8, 9). COPD patients may have a higher risk of developing AF, potentially related to chronic hypoxia, inflammatory responses, and heightened sympathetic activity (10). Conversely, AF may aggravate dyspnea and exercise intolerance in COPD patients and may be associated with worse prognosis (11, 12).

Although existing studies have explored the relationship between COPD and AF, most are unidirectional and fail to offer a comprehensive view of the interrelationship. This study aims to systematically assess the bidirectional risk association between COPD and AF, as well as their combined impact on prognosis, using a more comprehensive bidirectional design, larger sample sizes, and rigorous statistical methods. Overall, the findings of this study may provide additional evidence for the clinical management of comorbidities.

## 2 Methods

This study adhered to the Preferred Reporting Items for Systematic Reviews and Meta-Analyses (PRISMA) guidelines (13). The review protocol was registered with PROSPERO (CRD420251069785).

### 2.1 Literature search

A systematic literature search was conducted for studies published from the inception of PubMed, EMBASE, and Cochrane Library up to June 5, 2025, focusing on the association between COPD and AF (the search strategy is outlined in Supplementary Table 1). Additionally, the references of the included studies were reviewed to identify any relevant studies that may have been missed.

### 2.2 Inclusion and exclusion criteria

Inclusion criteria were as follows: (1) Cohort studies or case-control studies. (2) Studies reporting data on the association between COPD and AF, including the risk of COPD→AF, the risk of AF→COPD, the impact of AF on mortality in COPD patients, and the impact of COPD on mortality in AF patients.

In this study, “COPD→AF” refers to analyses in which COPD was the exposure or index condition and AF was the outcome, whereas “AF→COPD” refers to analyses in which AF was the exposure or index condition and COPD was the outcome. For prognostic analyses, “AF in COPD patients” and “COPD in AF patients” refer to the coexistence of the two conditions and do not imply a causal or temporal sequence.

(3) Clear diagnosis of COPD, based on pulmonary function test results or COPD diagnostic codes extracted from medical records. (4) Clear diagnosis of AF, based on electrocardiogram results or AF diagnosis codes extracted from medical records.

Exclusion criteria were as follows: (1) Duplicate articles, non-human studies, case reports, reviews, meta-analyses, or conference papers. (2) If multiple publications derived from the same cohort, the publication with the most recent data was included. (3) Studies for which the full text could not be obtained. (4) Non-English language articles. (5) Cohorts where both the study and control populations had other diseases that could affect the outcome (e.g., coronary heart disease, asthma).

### 2.3 Data extraction and quality assessment

Two authors (Wang and Ding) independently screened the abstracts and reviewed the full texts for eligibility, resolving any disputes by consensus with a third independent author (Liu). The following data were extracted from the included studies: authors, year, country, sample size, study design (prospective or retrospective cohort study), baseline characteristics of patients, type of COPD (COPD or AECOPD), and risk data (HR, RR, OR), which were unified as OR for pooling. Study quality was evaluated using the Newcastle-Ottawa Scale (NOS) (14), with studies scoring ≥7 considered high quality.

### 2.4 Data synthesis and analysis

This study extracted HR, RR, and OR from the included articles and quantified them uniformly as OR (15). A meta-analysis was conducted using random-effects or fixed-effects models to assess the relationship between COPD and AF, defining events within 1 year of follow-up as short-term risks and events occurring at 1 year or longer as long-term risks. To visually present the comprehensive analysis of the studies, forest plots were generated. The I^2^ statistic was used to assess statistical heterogeneity across the studies. If heterogeneity was significant (*P* < 0.1 or I^2^ > 50%), a random-effects model was applied; otherwise, a fixed-effects model was used (16). Sensitivity analysis was performed to assess whether the pooled results were significantly influenced by any single study. Funnel plots and Egger's test were employed to assess publication bias. If significant bias was detected, the trim-and-fill method was used to evaluate the impact on results (17). All analyses were conducted using STATA statistical software version 15.1.

## 3 Results

### 3.1 Literature screening

**Figure 1:**
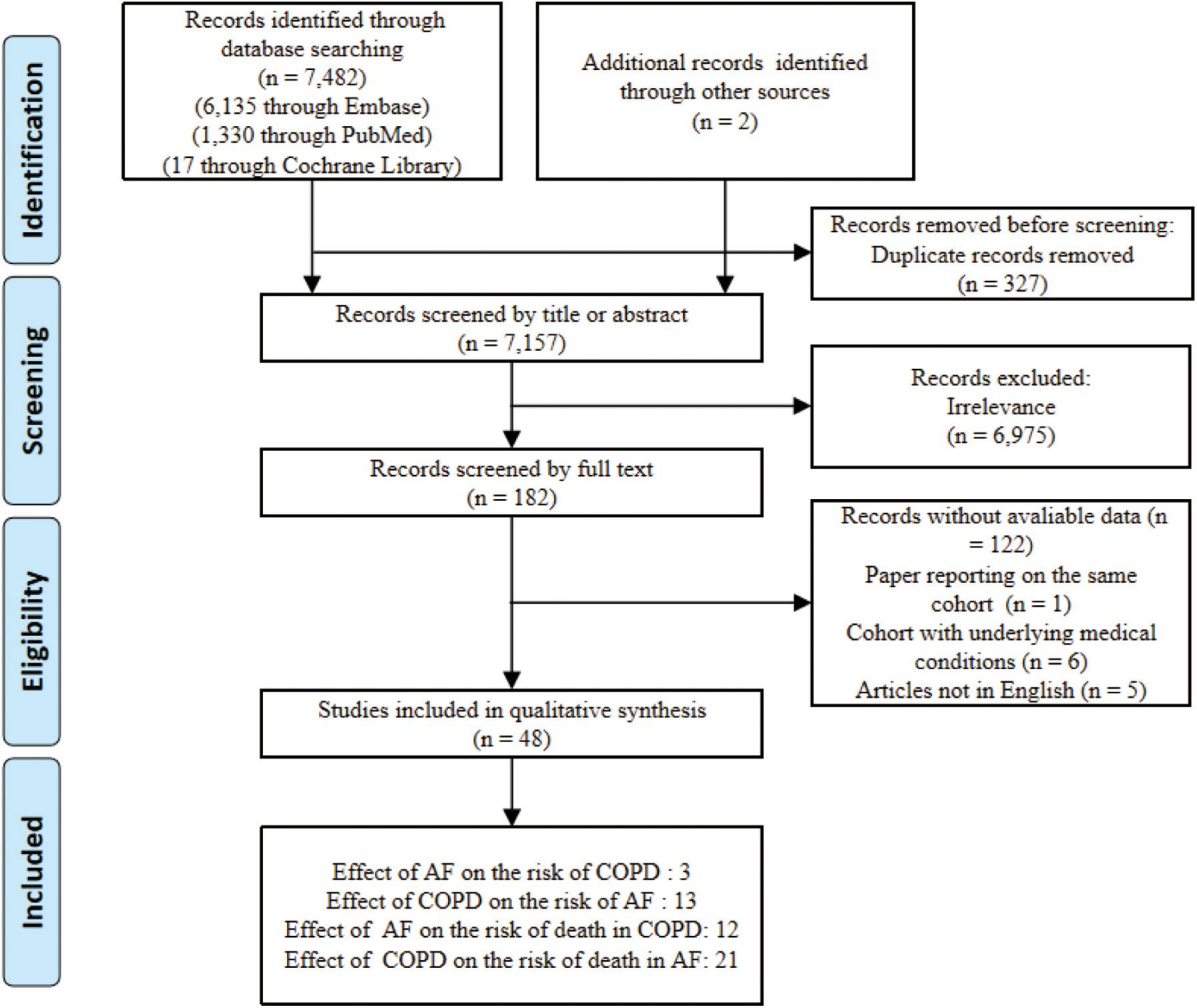
PRISMA flow diagram of the study selection process. Forty-eight unique publications were included. Carter et al. (2019) contributed data to two prespecified outcome analyses and was therefore counted once as a unique publication but twice across the outcome-specific categories; consequently, the sum of the outcome-specific study counts is 49.

A total of 7,484 articles were initially identified, including 7,482 through the search and 2 additional studies added from reference reviews. After excluding 327 duplicates, 7,157 articles were screened based on titles and abstracts, eliminating 6,975 irrelevant studies. The remaining 182 articles were thoroughly assessed, resulting in the inclusion of 48 unique publications that met the criteria. The study selection process is illustrated in Figure 1.

### 3.2 Study characteristics

Among the 48 included unique publications, 3 examined the risk of COPD in patients with AF (18–20), 13 investigated the risk of AF in patients with COPD (8, 21–32), 12 assessed the impact of AF on the prognosis of COPD patients [7 with in-hospital mortality as an endpoint (33–39), and 5 with follow-up periods exceeding 1 year (29, 40–43)], and 21 explored the impact of COPD on the prognosis of AF patients [4 with follow-up periods within 1 year (44–47), and 17 with follow-up periods exceeding 1 year (48–64)]. These outcome-specific categories were not mutually exclusive because Carter et al. (29) contributed data to two prespecified outcome analyses. For the AF→COPD analysis, three publications were included (18–20). Among them, Panaccio et al. (19) contributed two database-specific datasets, which were analyzed separately. The majority of the study populations were from Europe (8, 20–22, 24, 28–31, 33, 39, 42, 43, 46, 48, 50, 51, 53–58, 60, 62, 63), followed by North America and Asia. Most studies had sample sizes exceeding 1,000, although 8 studies on the impact of AF on the prognosis of COPD patients had sample sizes below 1,000 (33–35, 38–40, 42, 43).

The extent of covariate adjustment varied across the included studies. In the AF→COPD analysis, some studies adjusted for age, sex, and comorbidities, whereas others did not provide multivariable estimates. Across the disease-occurrence analyses, six studies relied solely on univariable estimates (19, 23, 28, 30–32). Among studies evaluating prognosis in patients with both AF and COPD, only one used univariable data (53).

### 3.3 Quality assessment

The quality of the included studies was assessed using the NOS, with the majority rated as high quality (scores ≥7.0), and only 4 studies considered of medium quality (scores 5–6) (23, 30, 31, 53). The quality scores for the studies are presented in Supplementary Tables 2–5.

### 3.4 Data analysis

#### 3.4.1 Risk of COPD in patients with AF

**Figure 2:**
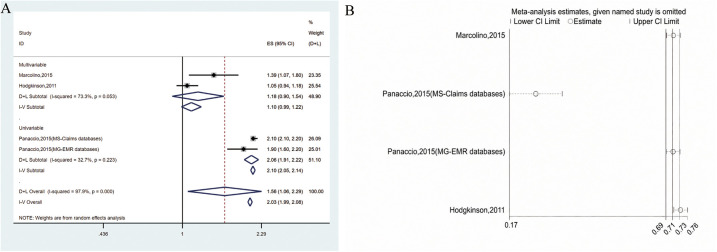
Comprehensive analysis of the risk of COPD occurrence in patients with AF. **(A)** Forest plot of the risk of COPD occurrence in patients with AF. **(B)** Sensitivity analysis of the risk of COPD occurrence in patients with AF.

In the three studies examining the risk of COPD in patients with AF, the study by Panaccio (2015) (19) provided two data sets from different databases. Random-effects model analysis indicated a significantly increased risk of COPD in patients with AF (OR 1.56, 95% CI: 1.06–2.29, I^2^ = 97.9%, *P* = 0.000). After excluding the Panaccio (2015) study, considerable heterogeneity persisted between the other two studies (Figure 2A). Sensitivity analysis showed that the Panaccio (2015) study (MS-Claims databases) notably influenced the final result (Figure 2B). Upon its exclusion, the pooled result was OR 1.29 (95% CI: 1.19–1.41, I^2^ = 94.4%, *P* = 0.000), still exhibiting significant heterogeneity. Due to the limited number of included studies, further exploration of the heterogeneity source was not possible. A funnel plot (Supplementary Figure 1) indicated some asymmetry, and Egger's test yielded a *P*-value of 0.287, suggesting no significant publication bias.

#### 3.4.2 Risk of AF in patients with COPD

**Figure 3:**
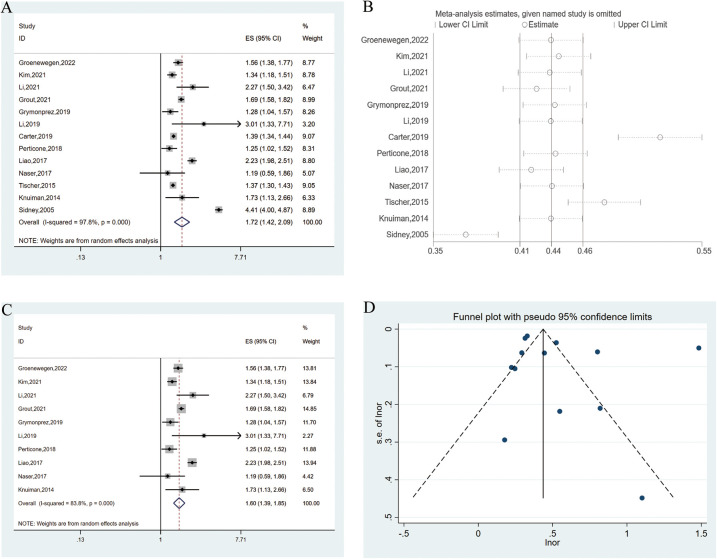
Comprehensive analysis of the risk of AF occurrence in patients with COPD. **(A)** Forest plot of the risk of AF occurrence in patients with COPD. **(B)** Sensitivity analysis of the risk of AF occurrence in patients with COPD. **(C)** Forest plot of the risk of AF occurrence in patients with COPD after excluding influential studies. **(D)** Funnel plot for publication bias of the risk of AF occurrence in patients with COPD.

This section included 13 studies. Random-effects model analysis demonstrated a significantly elevated risk of AF in patients with COPD (OR 1.72, 95% CI: 1.42–2.09, I^2^ = 97.8%, *P* = 0.000) (Figure 3A). Sensitivity analysis revealed that the studies by Carter (2019) (29), Tischer (2015) (31), and Sidney (2005) (32) had a significant impact on the results (Figure 3B). After excluding these three studies, the pooled result was OR 1.60 (95% CI: 1.39–1.85, I^2^ = 83.8%, *P* = 0.000) (Figure 3C), still exhibiting significant heterogeneity. Subgroup analysis based on region, analysis method, NOS score, and study design type did not clearly identify the source of heterogeneity. However, regional differences may explain some of the variation, as the pooled result from the 8 European studies was OR 1.39 (95% CI: 1.35–1.42, I^2^ = 38.5%, *P* = 0.123) (Supplementary Table 6), with lower heterogeneity. The funnel plot (Figure 3D) and Egger's test result (*P* = 0.359) indicated no significant publication bias.

#### 3.4.3 Impact of AF on the prognosis of COPD patients

##### 3.4.3.1 Impact of AF on the short-term prognosis of COPD patients

**Figure 4:**
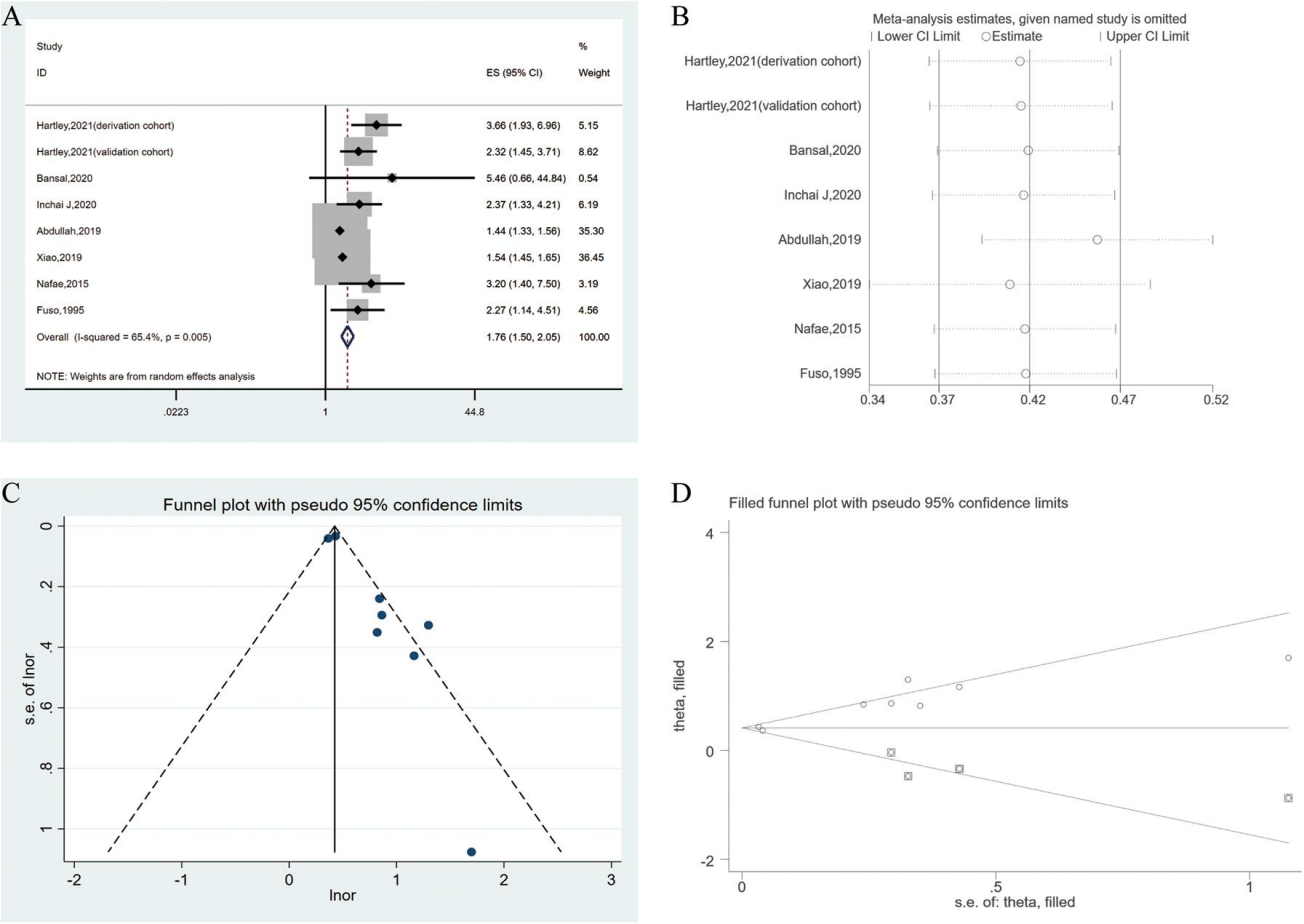
Comprehensive analysis of the impact of AF on the short-term prognosis of COPD patients. **(A)** Forest plot of the impact of AF on the short-term prognosis in COPD patients. **(B)** Sensitivity analysis of the impact of AF on the short-term prognosis in COPD patients. **(C)** Funnel plot for publication bias of the impact of AF on the short-term prognosis in COPD patients. **(D)** Trim-and-fill analysis for publication bias in assessing the impact of AF on short-term prognosis in COPD patients.

Eight studies from seven articles were included to investigate the relationship between AF and the short-term prognosis of COPD patients. Random-effects model analysis showed that AF was associated with a higher short-term mortality risk in COPD patients (OR 1.76, 95% CI: 1.50–2.05, I² = 65.4%, *P* = 0.005) (Figure 4A). Sensitivity analysis confirmed the robustness of the results, with no single study significantly affecting the pooled result (Figure 4B). Subgroup analysis based on region, analysis method, sample size, and study design suggested that sample size might be a key source of heterogeneity. When the sample size exceeded 1,000, the pooled OR was 1.50 (95% CI: 1.43–1.58, I^2^ = 39.2%, *P* = 0.200); when the sample size was smaller than 1,000, the pooled OR was 2.64 (95% CI: 2.02–3.46, I^2^ = 0.0%, *P* = 0.811) (Supplementary Table 7). The funnel plot showed asymmetry (Figure 4C), and Egger's test (*P* = 0.003) suggested the presence of publication bias. After adjusting for publication bias using the trim-and-fill method, the pooled OR was 1.59 (95% CI: 1.34–1.89), and the association remained significant after adjustment (Figure 4D).

##### 3.4.3.2 Impact of AF on the long-term prognosis of COPD patients

**Figure 5:**
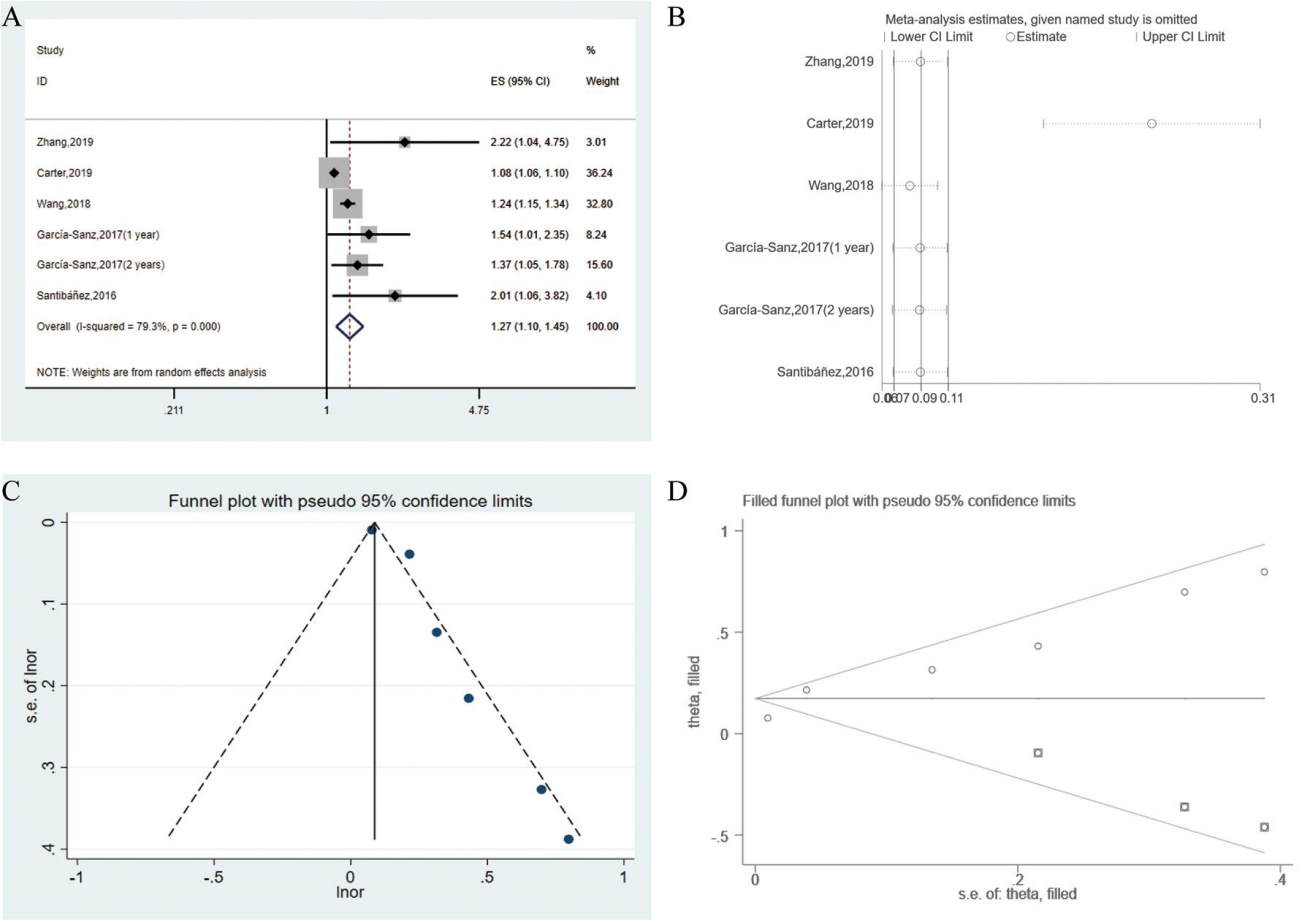
Comprehensive analysis of the impact of AF on the long-term prognosis of COPD patients. **(A)** Forest plot of the impact of AF on the long-term prognosis in COPD patients. **(B)** Sensitivity analysis of the impact of AF on the long-term prognosis in COPD patients. **(C)** Funnel plot for publication bias of the impact of AF on the long-term prognosis in COPD patients. **(D)** Trim-and-fill analysis for publication bias in assessing the impact of AF on long-term prognosis in COPD patients.

Six studies were included to analyze the association between AF and the long-term prognosis of COPD patients. Random-effects model analysis showed that AF was associated with a higher long-term mortality risk in COPD patients (OR 1.27, 95% CI: 1.10–1.45, I² = 79.3%, *P* = 0.000) (Figure 5A). Sensitivity analysis showed that the study by Carter (2019) (29) had a significant impact on the pooled result (Figure 5B). After excluding this study, the pooled OR was 1.27 (95% CI: 1.18–1.37), with reduced heterogeneity (I^2^ = 27.8%, *P* = 0.236). The source of heterogeneity was likely the Carter (2019) study, which may have been influenced by its large sample size and narrow 95% CI. The funnel plot exhibited some asymmetry (Figure 5C), and Egger's test (*P* = 0.01) indicated publication bias. After adjustment using the trim-and-fill method, the pooled OR was 1.26 (95% CI: 1.08–1.50), and the association remained significant after adjustment (Figure 5D).

#### 3.4.4 Impact of COPD on the prognosis of AF patients

##### 3.4.4.1 Impact of COPD on the short-term prognosis of AF patients

**Figure 6:**
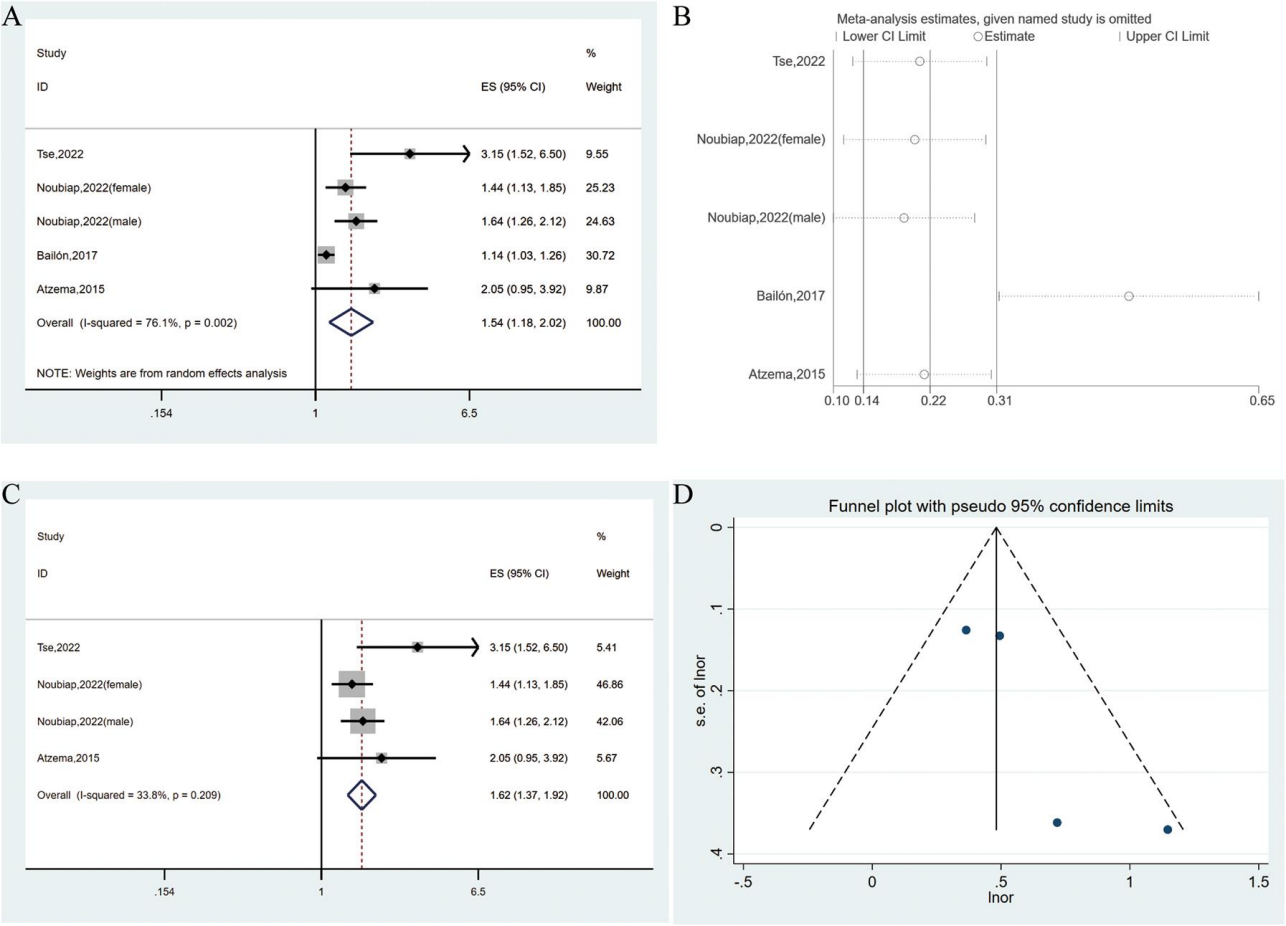
Comprehensive analysis of the impact of COPD on the short-term prognosis of AF patients. **(A)** Forest plot of the impact of COPD on the short-term prognosis in AF patients. **(B)** Sensitivity analysis of the impact of COPD on the short-term prognosis in AF patients. **(C)** Forest plot of the impact of COPD on the short-term prognosis in AF patients after excluding influential studies. **(D)** Funnel plot for publication bias of the impact of COPD on the short-term prognosis in AF patients.

Five studies from four articles were included to investigate the impact of COPD on the short-term prognosis of AF patients. Random-effects model analysis showed that COPD was associated with a higher short-term mortality risk in AF patients (OR 1.54, 95% CI: 1.18–2.02, I² = 76.1%, *P* = 0.002) (Figure 6A). Sensitivity analysis showed that the study by Bailón (2017) (46) had a significant influence on the results (Figure 6B). After excluding this study, fixed-effects model analysis showed a pooled OR of 1.62 (95% CI: 1.37–1.92, I^2^ = 33.8%, *P* = 0.209) (Figure 6C), indicating that the initial heterogeneity might primarily stem from this study. The funnel plot displayed slight asymmetry (Figure 6D), but Egger's test (*P* = 0.12) suggested no significant publication bias.

##### 3.4.4.2 Impact of COPD on the long-term prognosis of AF patients

**Figure 7:**
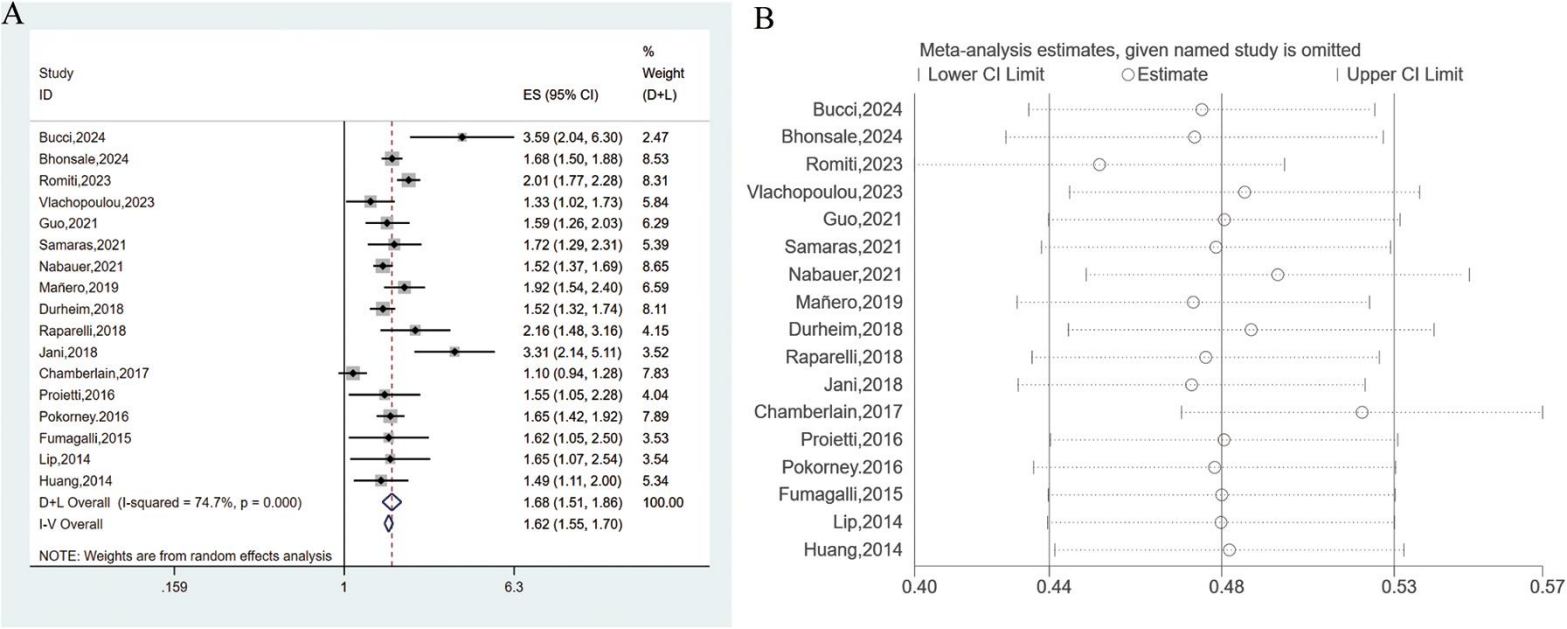
Comprehensive analysis of the impact of COPD on the long-term prognosis of AF patients. **(A)** Forest plot of the impact of COPD on the long-term prognosis in AF patients. **(B)** Sensitivity analysis of the impact of COPD on the long-term prognosis in AF patients.

Seventeen studies were included to analyze the impact of COPD on the long-term prognosis of AF patients. Random-effects model analysis showed that COPD was associated with a higher long-term mortality risk in AF patients (OR 1.68, 95% CI: 1.51–1.86, I² = 74.7%, *P* = 0.000) (Figure 7A). Sensitivity analysis indicated that the results were stable, with no single study significantly affecting the pooled result (Figure 7B). Although significant heterogeneity was present, subgroup analysis did not identify the source of the heterogeneity (Supplementary Table 8). As shown in Figure 7A, all studies indicated an increase in mortality risk, though the degree of increase varied. The funnel plot (Supplementary Figure 2) and Egger's test result (*P* = 0.272) suggested no significant publication bias.

## 4 Discussion

This study quantitatively assessed the epidemiological association and prognostic interaction between COPD and AF from a bidirectional perspective through systematic review and meta-analysis. The results showed an association with a higher risk of COPD in patients with AF (pooled OR 1.5). In the reverse association, the risk of AF in COPD patients was also increased (pooled OR 1.7). Notably, while the analysis of COPD→AF risk showed significant heterogeneity (I^2^ = 97.8%), sensitivity analysis revealed that the association remained after excluding outlier studies (OR 1.60). Additionally, heterogeneity was significantly reduced in the European population subgroup analysis (I^2^ = 38.5%, OR 1.39), suggesting that regional differences may be a key influencing factor. In terms of prognosis, the coexistence of COPD and AF was associated with worse prognosis in both directions: AF in COPD patients was associated with increased short-term (in-hospital mortality OR 1.76) and long-term mortality risks (OR 1.27). Similarly, COPD in AF patients was associated with increased short-term (OR 1.54) and long-term mortality risks (OR 1.68). Further confirmation through Egger's test and the trim-and-fill method indicated that these associations were not significantly affected by publication bias.

Previous observational studies have consistently identified smoking and advanced age as the strongest common risk factors for the comorbidity of AF and COPD (65). In the respiratory system, oxidative stress induced by tobacco's toxic components directly damages the airway epithelial barrier and accelerates lung parenchymal destruction through the proteinase-antiproteinase imbalance, resulting in a progressive decline in FEV_1_ (66, 67). In the cardiovascular system, smoking-induced coronary endothelial dysfunction (RR 1.32) and myocardial fibrosis substantially increase susceptibility to AF, with a clear dose-response relationship (for every additional 10 cigarettes smoked per day, the risk of AF increases by 14%) (68). Aging exacerbates the comorbidity risk through multiple pathological pathways, such as the degradation of elastic proteins in lung parenchyma leading to dynamic emphysema and degenerative changes in the cardiac conduction system, which enhance electrical activity heterogeneity and lower the threshold for AF occurrence (65).

The AF→COPD association requires more cautious interpretation than the COPD→AF association. Several mechanisms may plausibly explain how COPD contributes to AF, including hypoxaemia, pulmonary hypertension, systemic inflammation, autonomic imbalance, and atrial remodelling. In contrast, a direct pathophysiological pathway by which AF initiates fixed airflow obstruction or COPD is less clearly established. The observed association between AF and subsequent COPD may therefore partly reflect shared risk factors, particularly older age and smoking exposure, as well as cardiovascular comorbidities, residual confounding, surveillance bias, or misclassification of dyspnoea related to AF or heart failure as COPD. Given the very high heterogeneity in the AF→COPD analysis, this finding should be considered exploratory and hypothesis-generating rather than evidence of a causal effect.

Notably, in most of the study models included in the analysis, an independent association remained evident after adjusting for confounding factors such as smoking, age, and comorbidities. The Atherosclerosis Risk in Communities (ARIC) prospective cohort study in the United States demonstrated that COPD or a decline in lung function independently increased the risk of AF, regardless of smoking status. In never-smokers, those in the lowest quartile of FEV_1_ exhibited a significantly higher risk of AF, with the highest HR reaching 5.04. After adjusting for age, smoking status, and smoking quantity, airflow limitation (FEV_1_/FVC < 0.70) still increased the risk of AF by 37%–69% (69). This supports a possible pathophysiological link between the two conditions. The underlying mechanisms may involve three key factors: First, a decline in lung function (e.g., reduced FEV_1_) and airflow limitation (FEV_1_/FVC < 0.70) may lead to alveolar hypoventilation and hypoxemia, which may contribute to pulmonary hypertension and increased right ventricular afterload (70). This alteration in right ventricular function may affect left atrial structure and function via the interatrial septum, potentially contributing to left atrial dilation, increased wall stress, atrial myocyte fibrosis, and electrical remodeling. These pathological changes may provide a substrate for AF occurrence (71–73). Second, COPD patients often experience a systemic inflammatory response, marked by elevated inflammatory markers (such as C-reactive protein and interleukin-6) (74) and enhanced oxidative stress. These factors may further destabilize atrial myocytes electrically, promote the formation of ectopic pacemakers, and sustain micro-reentrant circuits, thereby contributing to the development and maintenance of AF (75). Finally, COPD patients may experience intermittent increases in atrial pressure and volume load due to frequent respiratory infections or inflammatory exacerbations, which also contribute to the development of AF (76).

The results of our meta-analysis also suggested an association between the coexistence of COPD and AF and worse patient prognosis in both directions. In COPD patients, the presence of AF was associated with increased short-term (in-hospital mortality OR 1.76) and long-term (OR 1.27) mortality risks. Several plausible mechanisms may partly contribute to this association, including hemodynamic impairment and amplification of inflammatory responses. The loss of effective atrial contraction and irregular ventricular rate (77) associated with AF may reduce cardiac output by approximately 20% (78), further exacerbating the existing ventilation/perfusion mismatch and hypoxemia in COPD patients (79, 80). Persistent hypoxia may activate the sympathetic nervous system and increase pulmonary vascular resistance, thereby contributing to pulmonary hypertension and further cardiopulmonary deterioration (81).

Conversely, in AF patients, the presence of COPD was also associated with increased short-term (OR 1.54) and long-term (OR 1.68) mortality risks. This association may be partly related to the effects of COPD on atrial structure and electrophysiology, as well as differences in treatment response. Chronic hypoxemia, a core pathological change in COPD, may activate the hypoxia-inducible factor-1*α* (HIF-1*α*) signaling pathway, promoting the proliferation of atrial fibroblasts and collagen deposition, and accelerating the fibrosis process of the atria (82, 83). This may provide an anatomical substrate for the induction and maintenance of AF. Additionally, the hypercapnia often accompanying COPD may increase the risk of cardiac electrical signal-triggered activity and reentrant circuit formation, thereby increasing the likelihood of AF combined with malignant arrhythmias (84). Moreover, inflammation plays a critical role. Inflammatory factors released by atrial myocytes in AF (such as CRP, fibrinogen) (85, 86) and the systemic inflammatory background of COPD (such as elevated IL-6, TNF-α) may interact and intensify the inflammatory response. Furthermore, the presence of COPD has been associated with reduced effectiveness of AF treatment, particularly in catheter ablation. Large cohort studies consistently show that patients with COPD have a significantly increased risk of AF recurrence following ablation surgery (87). Taken together, these plausible pathophysiological mechanisms may partly explain the worse prognosis observed in patients with both AF and COPD. However, because the included studies were observational, these mechanisms should be interpreted as potential contributors rather than proven causal pathways. In addition, patients with concomitant AF and COPD often represent a clinically complex phenotype, characterized by multimorbidity, polypharmacy, higher overall vulnerability, and more complicated clinical management (88). Such clinical complexity may independently contribute to mortality, cardiovascular events, bleeding, hospitalisation, and other adverse outcomes (51, 58, 88). Therefore, the observed prognostic associations likely reflect both disease-specific mechanisms and the broader burden of clinical complexity.

In clinical practice, managing patients with comorbid COPD and AF presents important therapeutic challenges, which may contribute to management complexity and poorer outcomes. On the one hand, the classic drug for controlling the ventricular rate of AF—*β*-blockers (e.g., propranolol, metoprolol)—can potentially block *β*2-receptors, which may induce bronchospasm, particularly in COPD patients (especially those with a tendency toward asthma or airway hyperresponsiveness), limiting their use (50, 89, 90). On the other hand, the core treatment for COPD—*β*2-receptor agonists (especially long-acting *β*2-receptor agonists)—carries potential arrhythmogenic risks, particularly increasing the incidence of atrial arrhythmias (such as AF), with a pooled OR of approximately 1.66 (91). Additionally, theophylline, still used in COPD management, has proarrhythmic properties (92). This “drug dilemma” forces clinicians to make difficult decisions: strictly controlling heart rate may induce bronchospasm and worsen respiratory failure, while expanding the airways may provoke or exacerbate arrhythmias. Although some studies report that *β*-blocker use is safe and not associated with worse pulmonary function outcomes (93), patients with COPD and AF are less likely to receive *β*-blocker therapy in practice, opting instead for drugs like digoxin to control the rate (50). However, digoxin has a narrow therapeutic window, a heightened risk of toxicity, and can lead to fatal arrhythmias (e.g., ventricular tachycardia, ventricular fibrillation) (94), especially in the elderly, patients with renal insufficiency, and those with hypoxia or electrolyte disturbances, creating another risk factor that threatens prognosis. This therapeutic dilemma may represent an additional contributor to poorer outcomes in patients with comorbid COPD and AF.

Therefore, for patients with comorbid COPD and AF, it is crucial to remain highly vigilant about potential drug antagonism and the additive adverse effects when formulating treatment plans, conducting meticulous individualized assessments and selections. To control the ventricular rate of AF while minimizing the risk of bronchospasm, high cardioselective *β*_1_-receptor blockers (e.g., bisoprolol) (95) can be considered, or non-dihydropyridine calcium channel blockers (e.g., diltiazem or verapamil) (1, 96) may be used if hemodynamics permit. In treating COPD, prioritizing the use of long-acting anticholinergic drugs (e.g., tiotropium, glycopyrronium) or employing combination formulations containing anticholinergic drugs (e.g., LABA/LAMA) is an important strategy to reduce the risk of arrhythmias (97). Simultaneously, if digitalis drugs are used, close monitoring of serum drug concentrations and electrolytes is essential.

## 5 Limitations

This study has several limitations. First, COPD-related and AF-related drugs may significantly impact patient prognosis; however, most studies included in our analysis did not clearly document the specific drugs used by patients during follow-up, and the impact of drug use was not adjusted for in the statistical analyses. Second, the diagnosis of COPD in our study primarily relied on diagnostic codes from medical databases, which may not accurately reflect the severity of the disease. Furthermore, in most of the studies included, the GOLD classification of COPD was not adjusted for in the multivariate regression models. Some studies have indicated that patients with severely impaired lung function (FEV_1_% < 50) have a higher risk of developing AF compared to those with mildly impaired lung function (50 ≤ FEV_1_% ≤ 70) (70), and this difference may have introduced bias into the final results. Third, substantial heterogeneity was observed in several pooled analyses. This may be related to differences in study design, population characteristics, diagnostic criteria for COPD and AF, follow-up duration, outcome definitions, and covariate adjustment. In particular, for the AF→COPD association, the number of included studies was limited and the pooled estimate showed very high heterogeneity. Because only a small number of studies contributed to this analysis, further subgroup analyses or meta-regression to explore potential sources of heterogeneity were not feasible. Therefore, especially for the AF→COPD direction, the pooled estimate should be interpreted cautiously. Finally, because all included studies were observational, causality could not be inferred. Residual confounding related to multimorbidity, frailty, disease severity, medication use, and overall clinical complexity may have influenced the observed associations.

## 6 Conclusions

This study suggests a bidirectional observational association between COPD and AF, as well as an association between their coexistence and increased mortality risk. However, given the observational nature of the included studies, substantial heterogeneity, and the clinical complexity of patients with coexisting COPD and AF, these findings should be interpreted as associations rather than causal effects. These results highlight the importance of vigilant screening for the other condition in patients diagnosed with either COPD or AF, and support integrated, individualized management strategies for comorbid patients. When selecting treatment plans, careful consideration should be given to potential drug interactions and competing cardiopulmonary risks to optimize patient prognosis.

## Data Availability

The original contributions presented in the study are included in the article/Supplementary Material, further inquiries can be directed to the corresponding author.
