# Supplementary material for: Bidirectional association between COPD and AF: a systematic review and meta-analysis
Source: Front Cardiovasc Med. 2026 Jun 19;13:1840570. doi: 10.3389/fcvm.2026.1840570 (PMC13327927; doi:10.3389/fcvm.2026.1840570)

Supplementary Material

# Supplementary Tables

| Supplementary Table 1. The details of search strategies. | | | |
| --- | --- | --- | --- |
| Database | Search step | Search strategy | Search result |
| PubMed |  |  |  |
|  | #1 | ((((((((((((((((((((((((Atrial Fibrillations) OR (Fibrillation, Atrial)) OR (Fibrillations, Atrial)) OR (Auricular Fibrillation)) OR (Auricular Fibrillations)) OR (Fibrillation, Auricular)) OR (Fibrillations, Auricular)) OR (Persistent Atrial Fibrillation)) OR (Atrial Fibrillation, Persistent)) OR (Atrial Fibrillations, Persistent)) OR (Fibrillation, Persistent Atrial)) OR (Fibrillations, Persistent Atrial)) OR (Persistent Atrial Fibrillations)) OR (Familial Atrial Fibrillation)) OR (Atrial Fibrillation, Familial)) OR (Atrial Fibrillations, Familial)) OR (Familial Atrial Fibrillations)) OR (Fibrillation, Familial Atrial)) OR (Fibrillations, Familial Atrial)) OR (Paroxysmal Atrial Fibrillation)) OR (Atrial Fibrillation, Paroxysmal)) OR (Atrial Fibrillations, Paroxysmal)) OR (Fibrillation, Paroxysmal Atrial)) OR (Fibrillations, Paroxysmal Atrial)) OR (Paroxysmal Atrial Fibrillations) | 122,390 |
|  |  |  |  |
|  | #2 | (((((((((Chronic Obstructive Pulmonary Diseases) OR (COPD)) OR (Chronic Obstructive Lung Disease)) OR (Chronic Obstructive Pulmonary Disease)) OR (COAD)) OR (Chronic Obstructive Airway Disease)) OR (Airflow Obstruction, Chronic)) OR (Airflow Obstructions, Chronic)) OR (Chronic Airflow Obstructions)) OR (Chronic Airflow Obstruction) | 125,425 |
|  |  |  |  |
|  | #3 | #1 AND #2 | 1,470 |
|  |  |  |  |
|  | #4 | review[Publication Type] | 3,662,804 |
|  |  |  |  |
|  | #5 | #3 NOT #4 | 1,330 |
|  |  |  |  |
|  |  |  |  |
| Embase |  |  |  |
|  | #1 | 'atrial fibrillation'/exp OR 'atrial fibrillation' | 267,422 |
|  |  |  |  |
|  | #2 | 'chronic obstructive lung disease'/exp OR 'chronic obstructive lung disease' | 205,388 |
|  |  |  |  |
|  | #3 | [article]/lim | 31,511,261 |
|  |  |  |  |
|  | #4 | #1 AND #2 AND #3 | 6,135 |
|  |  |  |  |
| Cochrane Library |  |  |  |
|  | #1 | MeSH descriptor: [Pulmonary Disease, Chronic Obstructive] explode all trees | 7,418 |
|  |  |  |  |
|  | #2 | MeSH descriptor: [Atrial Fibrillation] explode all trees | 7,982 |
|  |  |  |  |
|  | #3 | #1 AND #2 | 17 |
|  |  |  |  |

| Supplementary Table 2. Characteristics of studies on the risk of COPD in AF included in this meta-analysis. | | | | | | | | | | |
| --- | --- | --- | --- | --- | --- | --- | --- | --- | --- | --- |
| Author, year | Country | Population, n | Female, % | Age | Study design | Follow-up (year) | COPD diagnosis | AF diagnosis | Multivariable analysis | NOS |
| Marcolino,2015 | Brazil | 262685 | 59.6 | 50.3 ± 19.3 | Retrospective cohort | NA | self-reported | ECG | Age and sex | 7 |
| Panaccio,2015 | USA (MS-Claims databases) | 210524 | 50.8 | 74 | Prospective cohort | 1.7-1.9years | Spirometry | Based on ICD codes | NA | 6 |
| Panaccio,2015 | USA (MG-EMR databases) | 7791 | 45.2 | 72 | Prospective cohort | 1.9-2.0years | Spirometry | Based on ICD codes | NA |  |
| Hodgkinson,2011 | UK | 271812 | 49.7 | 73.8±11.8 | Retrospective cohort | NA | Spirometry | ECG | Age, sex and comorbidities | 7 |
| NA: Not available; NOS: Newcastle-Ottawa Scale | | | | | | | | | | |

| Supplementary Table 3. Characteristics of studies on the risk of AF in COPD included in this meta-analysis. | | | | | | | | | | |
| --- | --- | --- | --- | --- | --- | --- | --- | --- | --- | --- |
| Author, year | Country | Population, n | Female, % | Age | Study design | Follow-up (year) | COPD diagnosis | AF diagnosis | Multivariable analysis | NOS |
| Groenewegen,2022 | Netherlands | 143251 | 50.4 | 55.7±10.7 | Prospective cohort | 3.9 years | Based on ICPC codes | Based on ICPC codes | NA | 6 |
| [Kim,2021](https://pubmed.ncbi.nlm.nih.gov/?sort=date&term=Kim+IS&cauthor_id=34793457) | Korea | 501668 | 50 | 47.6 ± 14.3 | Prospective cohort | 46.2±15.1 months | Based on ICD codes | Based on ICD codes | age,sex,previous ischemic stroke or TIA, previous myocardial infarction, heart failure, hypertension,diabetes mellitus,CKD,obesity, smoking,excessive alcohol intake,and low physical activity. | 8 |
| Li,2021 | China | 23523 | 53.9 | 52.5±18.2 | Prospective cohort | 2.84±3.56 years | Based on ICD codes | ECG or 24h Holter | Age,sex and comorbidities | 8 |
| Grout,2021 | USA | 53552 | 54 | 66.5 | Retrospective cohort | 2 years | Based on ICD codes | Based on ICD codes | NA | 5 |
| Grymonprez,2019 | Netherlands | 10943 | 57.5 | 63.1 | Prospective cohort | 6.8 years | Spirometry | ECG | Age,sex and pack years of cigarette smoking. | 7 |
| Li,2019 | China | 471446 | 47.3 | 47.0±16 | Retrospective cohort | 11 years | Based on ICD codes | Based on ICD codes | Age,sex,comorbidities,and CHA2DS2-VASc score. | 9 |
| Carter,2019 | UK | 189876 | 59 | 70.1±12.0 | Retrospective cohort | 5.2 ± 3.6 years | Based on ICD codes | Based on ICD and OPCS-4 codes | Age,sex,race/ethnicity,and comorbidities | 7 |
| Perticone,2018 | Italy | 4322 | 47.5 | 59.2±11.7 | Prospective cohort | 59.4±22.7 months | Spirometry | ECG or 24h Holter | Age,gender,PP,BMI,smoking,hs-CRP，e-GFR and CHA2DS2-VASc score. | 7 |
| Liao,2017 | China | 18617 | 25 | 62 | Retrospective cohort | 15 years | Based on ICD codes | ECG | Age,urbanization,gender,and comorbidities. | 7 |
| Naser,2017 | Bosnia and Herzegovina | 2352 | 48 | 68.0±13.0 | Prospective cohort | 9.7 ± 1.8 years | Spirometry | ECG or 24h Holter | NA | 5 |
| Tischer,2015 | Germany | 150408 | 43.1 | 67.6±13.6 | Retrospective cohort | NA | Based on ICD codes | ECG | NA | 5 |
| Knuiman,2014 | Australia | 4267 | 56.4 | 52±15 | Prospective cohort | 15 years | Based on ICD codes | Based on ICD codes | Age,sex,,height, hypertension treatment and BMI terms. | 7 |
| Sidney,2005 | USA | 91932 | 44.6 | 64.4±12.2 | Retrospective cohort | 2.75-2.99 years | Based on ICD codes | Based on ICD codes | NA | 7 |
| NA: Not available; NOS: Newcastle-Ottawa Scale | | | | | | | | | | |

| Supplementary Table 4. Characteristics of studies on the impact of AF on COPD outcomes included in this meta-analysis. | | | | | | | | | | |  |
| --- | --- | --- | --- | --- | --- | --- | --- | --- | --- | --- | --- |
| Author, year | Country | Population, n | Female, % | Age | Study design | Follow-up (year) | COPD type | COPD diagnosis | AF diagnosis | Multivariable analysis | NOS |
| Hartley,2021(derivation cohort) | UK | 489 | 62.6 | 72.8±10.0 | Retrospective cohort | In hospital | AECOPD | Based on ICD codes | Based on ICD codes | Age, sex, smoking,BMI,Laboratory examination,medications and comorbidities. | 8 |
| Hartley,2021(validation cohort) | UK | 733 | 58.3 | 70.5±9.3 | Prospective cohort | In hospital | AECOPD | Spirometry | ECG | Age, sex, smoking,BMI,Laboratory examination,medications and comorbidities. | 9 |
| Bansal,2020 | India | 228 | 26.3 | 61.09 ± 10.6 | Retrospective cohort | In hospital | AECOPD | Spirometry | ECG | Age,sex,smoking,BMI,exacerbations/year,dyspnea,eosinopenia,consolidation,acidemia,and DECAF. | 7 |
| Inchai J,2020 | Thailand | 739 | 42 | 74.2±9.7 | Retrospective cohort | In hospital | COPD | Spirometry | ECG | Age,sex,coronary artery disease, depression, and COTE. | 7 |
| Abdullah,2019 | USA | 1377795 | 55.5 | 68.57 (68.52 - 68.61) | Retrospective cohort | In hospital | AECOPD | Based on ICD codes | Based on ICD codes | age,sex,cardiovascular risk factors,mechanical ventilation, acute kidney injury, and the presence of pneumonia or sepsis. | 9 |
| Xiao,2019 | China | 1345270 | 57.5 | 70.63±23.49 | Retrospective cohort | In hospital | AECOPD | Based on ICD codes | Based on ICD codes | Age,race,sex,income,insurance status,year,hospital region,hospital location/teaching status,  hospital bed size, and comorbidities. | 7 |
| Nafae,2015 | Egypt | 200 | 49 | 69.3 ± 8 | Prospective cohort | In hospital | AECOPD | Spirometry | ECG | Age,sex,FEV1,BMI,PH,exacerbations/year,Laboratory examination,and comorbidities. | 7 |
| Fuso,1995 | Italy | 590 | 21.4 | 67.9±10.2 | Retrospective cohort | In hospital | AECOPD | Spirometry | ECG | Age,PaO₂,PaCO₂,pH,PA-aO₂,medications and comorbidities. | 7 |
| [Zhang，2019](https://pubmed.ncbi.nlm.nih.gov/?sort=date&term=Zhang+Y&cauthor_id=31826532) | China | 890 | 29.3 | 73±10 | Prospective cohort | 41±17 months | AECOPD | Spirometry | ECG | Age,BMI,length of hospital stay,high eosinophil count,neutrophil percentage,lymphocyte count,hemoglobin concentration,albumin concentration,partial pressure of carbon dioxide,HF,ischemic cerebrovascular disease,lung cancer,chronic pulmonary heart disease,admission to ICU,non-invasive mechanical ventilation,invasive mechanical ventilation. | 7 |
| Carter,2019 | UK | 189876 | 59 | 70.1±12.0 | Retrospective cohort | 5.2 ± 3.6 years | COPD | Based on ICD codes | Based on ICD and OPCS-4 codes | Age,sex,race/ethnicity,and comorbidities. | 7 |
| Wang,2018 | China | 12438 | 27 | 71.2±9.0 | Retrospective cohort | 3.63 years | COPD | Based on ICD codes | Based on ICD codes | The time from COPD diagnosis to index date,age,gender, index year of AF,monthly income,hospital level,severe exacerbations of COPD in one year prior to index date (never, 1, or ≥2 times/year), medications for COPD,medications for hypertension,other medications,individual comorbidities,cardioversion Procedure and amiodarone use. | 9 |
| García-Sanz,2017 | Spain | 757 | 23 | 74.8±11.2 | Retrospective cohort | 1-5 years | AECOPD | Spirometry | ECG | Age,GOLD,hypertension,ischemic heart disease,cerebrovascular accident,dementia,chronic kidney disease,Charlson,O2 therapy and hospitalization due to AECOPD-previous year. | 7 |
| Santibáñez,2016 | Spain | 900 | 21.6 | 71.2±11.0 | Retrospective cohort | 2 years | COPD | Spirometry | ECG | Age, sex, smoking status, COPD severity (GOLD Grades 1–4) and frequent exacerbator phenotype (yes/no) the previous year. | 8 |
| NA: Not available; NOS: Newcastle-Ottawa Scale | | | | | | | | | | | |

| Supplementary Table 5. Characteristics of studies on the impact of COPD on AF outcomes included in this meta-analysis.. | | | | | | | | | | |  |
| --- | --- | --- | --- | --- | --- | --- | --- | --- | --- | --- | --- |
| Author, year | Country | Population, n | Female, % | Age | Study design | Follow-up (year) | COPD type | COPD diagnosis | AF diagnosis | Multivariable analysis | NOS |
| Tse,2022 | China | 4003 | 34.4 | 68.5 ± 11.8 | Prospective cohort | 364 days | COPD | Spirometry | ECG | Age,gender,BMI,diastolic blood pressure,AF as primary reason for admission/consultation,hypertension,Diabetes mellitus,hyperlipidemia,previous heart failure,previous CAD,previous coronary intervention (PCI/CABG),previous valvular disease,hemorrhagic event,malignancy,diuretics and statins. | 7 |
| Noubiap,2022 | USA | 82592 | 50.8 | 70.6±12.9 | Retrospective cohort | In hospital | COPD | Based on ICD codes | Based on ICD codes | Age,sex (for the analysis on the entire population),race,median household income,comorbidities, tobacco smoking and alcohol abuse,and rhythm control procedures (catheter ablation or electrical cardioversion). | 7 |
| Bailón,2017 | Spain | 210605 | 52.7 | 73.45 | Retrospective cohort | In hospital | COPD | Based on ICD codes | Based on ICD codes | Age,sex,Charlson Comorbidity Index,emergency room admission,readmissions <30 days,year of admission. | 8 |
| Atzema,2015 | Canada | 2343 | 49.7 | 68.8±14.7 | Retrospective cohort | 30 days | COPD | Based on ICD codes | Based on ICD codes | Age,systolic blood pressure,heart rate,chest pain,positive troponin,creatinine level > 200 mmol/L,smoker status,cancer，dementia，bleeding risk，other acute ED diagnosis. | 7 |
| Bucci,2024 | UK | 4094 | 34.6 | 68.5±11.9 | Prospective cohort | 1 year | COPD | Spirometry | ECG | Age, sex, paroxysmal atrial fibrillation, CHA2DS2‐VASc score, chronic kidney disease, cancer, dyslipidemia, dementia, oral anticoagulation, and beta blocker use. | 8 |
| Bhonsale,2024 | USA | 17335 | 32.5 | 56.1±8.01 | Retrospective cohort | >5 years | COPD | Based on ICD codes | Based on ICD codes | Age,gender,race,cardiac interventions,smoking history,morbid obesity,cardiovascular risk factors,CKD,liver disease,history of cancer and medication use. | 8 |
| Romiti,2023 | UK | 36263 | 45.2 | 70.1 ± 10.5 | Prospective cohort | 2 years | COPD | Spirometry | ECG | Age, sex, geographical location, race/ethnicity, CHA2DS2-VASc score, type of AF, CAD, heart failure, phase of recruitment, treatment with OAC, and beta-blocker. | 8 |
| Vlachopoulou,2023 | Greece | 1130 | 44.5 | 76.1 | Prospective cohort | 2.7 years | COPD | Prescription for inhalation therapy | ECG | Age, BBs, diabetes mellitus, left ventricular ejection fraction (LVEF) ≤ 40%, and history of coronary artery disease (CAD) as well as prior stroke. | 8 |
| Guo,2021 | China | 5474 | 39.7 | 73.4 ± 10.6 | Prospective cohort | 1 year | COPD | Spirometry | ECG | Sex, hypertension, coronary artery disease, liver dysfunction, and prior major bleeding. | 7 |
| Samaras,2021 | Greece | 1130 | 45.3 | 76(68-81) | Prospective cohort | 2 years | COPD | Spirometry | ECG | NA | 7 |
| Nabauer,2021 | Germany | 8830 | 38.4 | 68.2±10.8 | Prospective cohort | 6.46 years | COPD | Spirometry | ECG | Age,CKD, diabetes mellitus, BMI, malignancy, smoking, NYHA class, left ventricular systolic dysfunction in transthoracic echocardiography,hypertension, peripheral artery disease, , prior stroke/TIA, prior systemic embolism, pulmonary embolism, type of AF,and haemorrhagic complication. | 7 |
| Mañero，2019 | Spain | 7990 | 50.8 | 76.8±10.5 | Prospective cohort | 707±103 days | COPD | Spirometry | ECG | Age gender, CHA2DS2-VASc score, valvular and non-valvular AF, peripheral arteriopathy, diabetes mellitus (insulin-dependent or non-insulin dependent), arterial hypertension, pulmonary thromboembolism, ischemic transitory accident/stroke, heart failure, acute myocardial infarction, death (inpatient and outpatient) or treatment with warfarin/acenocumarol, aspirin, clopidogrel, rivaroxaban, apixaban or dabigatran, beta-blockers, angiotensin-converting enzyme inhibitors/angiotensin II receptor antagonist (ACEI/ARAII), and digoxin. | 7 |
| Durheim,2018 | Norway | 9749 | 42.6 | 75 | Prospective cohort | >2 years | COPD | Spirometry | ECG | Age gender,smoking status,type of AF,duration of AF,LVEF,Left atrial diameter type,Treatment-related factors and Comorbidities. | 8 |
| Raparelli,2018 | Italy | 2027 | 45 | 73 ± 10 | Prospective cohort | 3 years | COPD | Spirometry | ECG | Gender, hypertension, diabetes mellitus, dyslipidemia,  smoking habit, prior Stroke/TIA, beta-blockers, ACEs, digoxin, creatinine clearance, HF, statins, ARBs, and anti dysrhythmic agents. | 8 |
| Jani,2018 | UK | 3651 | 31.7 | 61.9±6 | Prospective cohort | 7 years | COPD | Spirometry | ECG | Age, sex, socio-economic, smoking, and anticoagulation status. CHD, coronary heart disease; | 7 |
| Chamberlain,2017 | USA | 1430 | 51.4 | 73.6±13.8 | Prospective cohort | 6.3±3.9 | COPD | Based on ICD codes | Based on ICD codes | Age,sex,and all other comorbidities. | 8 |
| Proietti,2016 | UK | 3119 | 39.9 | 68.7±11.5 | Prospective cohort | 1 year | COPD | Spirometry | ECG | Age gender,smoking status,type of AF,LVEF,CHA2DS2-VASc Score,Thromboembolic Risk, Thromboembolic Risk,Treatment-related factors and Comorbidities. | 7 |
| Pokorney.2016 | USA | 14171 | 39.5 | 73 | Prospective cohort | 1.9 years | COPD | Spirometry | ECG | Age, sex, race, ethnicity (Hispanic or non‐Hispanic), region, heart rate, body mass index, systolic blood pressure, diastolic blood pressure, years since AF diagnosis, type of AF (persistent, paroxysmal, recent onset), prior stroke or transient ischemic attack, heart failure, hypertension, diabetes mellitus, creatinine clearance, creatinine, peripheral arterial disease, prior gastrointestinal bleeding, liver disease, alcohol use, obstructive sleep apnea, and left bundle branch block. | 9 |
| Fumagalli,2015 | Italy | 3119 | 40.4 | 69 ± 11 | Prospective cohort | 366 ± 32 days | COPD | Spirometry | ECG | Age,malignancy, history of bleeding events, and previous transient ischaemic attack (TIA),other comorbidities,oral anticoagulant,antiplatelet drug,Biomarkers and laboratory tests and treatments and interventions. | 7 |
| Lip，2014 | UK | 2589 | 39.4 | 68.7+11.6 | Prospective cohort | 366 days | COPD | Spirometry | ECG | Age, AF as reason for admission/ consultation, previousTIA, chronic kidney disease, malignancy , bleeding, diuretics, Statins. | 7 |
| Huang,2014 | China | 1975 | 45.2 | 68.37±13.3 | Prospective cohort | 1 year | COPD | Spirometry | ECG | Age,sex,weight,medical history (type of AF, myocardial infarction, hypertension, diabetes mellitus,heart failure,valve disease,smoking,stroke,or TIA),blood pressure,heart rate,and main medications including warfarin,  aspirin,clopidogrel,beta-blocker,digoxin,calcium-channel blockers,cardioversion drugs(amiodarone,sotalol,and propafenone),diuretic, angiotensin-converting enzyme inhibitor,as well as other treatment strategies (electrical cardioversion, catheter ablation). | 7 |
| NA: Not available; NOS: Newcastle-Ottawa Scale | | | | | | | | | | | |

| Supplementary Table 6. Subgroup analysis of studies on COPD and the risk of AF among all included studies. | | | | | |
| --- | --- | --- | --- | --- | --- |
| Variables | No. of studies | OR (95%CI) | Heterogeneity | | Model |
|  |  |  | I^2^ (%) | P |  |
| **Area** |  |  |  |  |  |
| America | 2 | 2.73 (1.07 - 6.98) | 99.6 | <0.001 | Random |
| Europe | 8 | 1.39 (1.35 - 1.42) | 38.5 | 0.123 | Fixed |
| Australia | 1 | 1.73 (1.13 - 2.66) | - | - | - |
| Asia | 2 | 2.24 (1.99 - 2.52) | 0.0 | 0.507 | Fixed |
| **Analysis** |  |  |  |  |  |
| M | 5 | 1.84 (1.20 - 2.84) | 99.1 | <0.001 | Random |
| U | 8 | 1.59 (1.33 - 1.90) | 89.8 | <0.001 | Random |
| **NOS** |  |  |  |  |  |
| ≤ 6 | 4 | 1.51 (1.31 - 1.74) | 87.9 | <0.001 | Random |
| > 6 | 9 | 1.87 (1.31 - 2.67) | 98.5 | <0.001 | Random |
| **Study designs** |  |  |  |  |  |
| Retrospective cohort | 6 | 2.05 (1.51 - 2.79) | 99.1 | <0.001 | Random |
| Prospective cohort | 7 | 1.42 (1.32 - 1.53) | 48.9 | 0.068 | Fixed |
| AF: atrial fibrillation; COPD: chronic obstructive pulmonary disease; M Multivariable; U Univariable; NOS Newcastle-Ottawa Scale | | | | | |

| Supplementary Table 7. Subgroup analysis of studies on the short-term prognosis of COPD associated with AF among all included studies. | | | | | |
| --- | --- | --- | --- | --- | --- |
| Variables | No. of studies | OR (95%CI) | Heterogeneity | | Model |
|  |  |  | I^2^ (%) | P |  |
| **Area** |  |  |  |  |  |
| America | 1 | 1.44 (1.33 - 1.56) | - | - | - |
| Europe | 3 | 2.61 (1.87 - 3.63) | 0.0 | 0.480 | Fixed |
| Africa | 1 | 3.20 (1.40 - 7.50) | - | - | - |
| Asia | 3 | 1.55 (1.45 - 1.65) | 42.8 | 0.174 | Fixed |
| **Sample size** |  |  |  |  |  |
| > 1000 | 2 | 1.50 (1.43 - 1.58) | 39.2 | 0.200 | Fixed |
| < 1000 | 6 | 2.64 (2.02 - 3.46) | 0.0 | 0.811 | Fixed |
| **Study designs** |  |  |  |  |  |
| Retrospective cohort | 6 | 1.63 (1.41 - 1.89) | 64.6 | 0.015 | Random |
| Prospective cohort | 2 | 2.50 (1.66 - 3.77) | 0.0 | 0.512 | Fixed |
| AF: atrial fibrillation; COPD: chronic obstructive pulmonary disease; M Multivariable; U Univariable; NOS Newcastle-Ottawa Scale | | | | | |

| Supplementary Table 8. Subgroup analysis of studies on the long-term prognosis of AF associated with COPD among all included studies. | | | | | |
| --- | --- | --- | --- | --- | --- |
| Variables | No. of studies | OR (95%CI) | Heterogeneity | | Model |
|  |  |  | I^2^ (%) | P |  |
| **Area** |  |  |  |  |  |
| America | 3 | 1.50 (1.39 - 1.62) | 90.4 | <0.001 | Random |
| Europe | 12 | 1.79 (1.58 - 2.04) | 69.2 | <0.001 | Random |
| Asia | 2 | 1.55 (1.29 - 1.87) | 0.0 | 0.740 | Fixed |
| **Analysis** |  |  |  |  |  |
| M | 16 | 1.68 (1.50 - 1.87) | 76.2 | <0.001 | Random |
| U | 1 | 1.72 (1.29 - 2.30) | - | - | - |
| **Study designs** |  |  |  |  |  |
| Retrospective cohort | 1 | 1.68 (1.50 - 1.88) | - | - | - |
| Prospective cohort | 16 | 1.68 (1.50 - 1.89) | 76.1 | <0.001 | Random |
| AF: atrial fibrillation; COPD: chronic obstructive pulmonary disease; M Multivariable; U Univariable; NOS Newcastle-Ottawa Scale | | | | | |

**Supplementary Figure 1**: Funnel plot for publication bias of the risk of COPD occurrence in patients with AF.


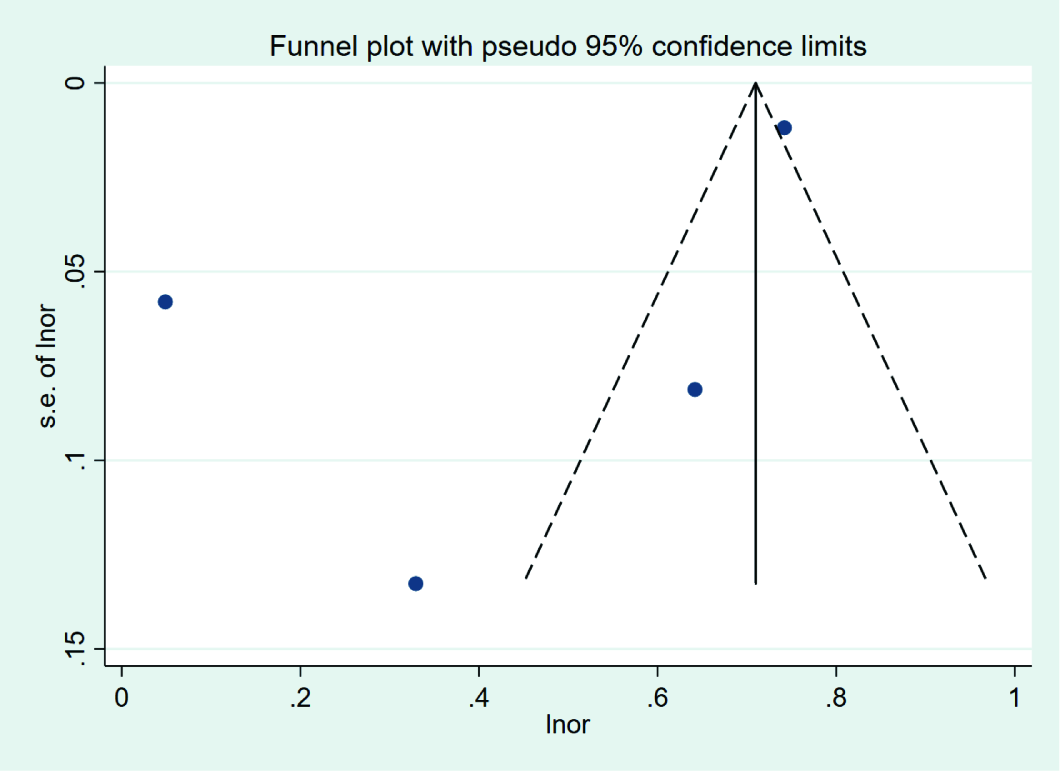


**Supplementary Figure 2**: Funnel plot for publication bias of the impact of COPD on the long-term prognosis in AF patients.


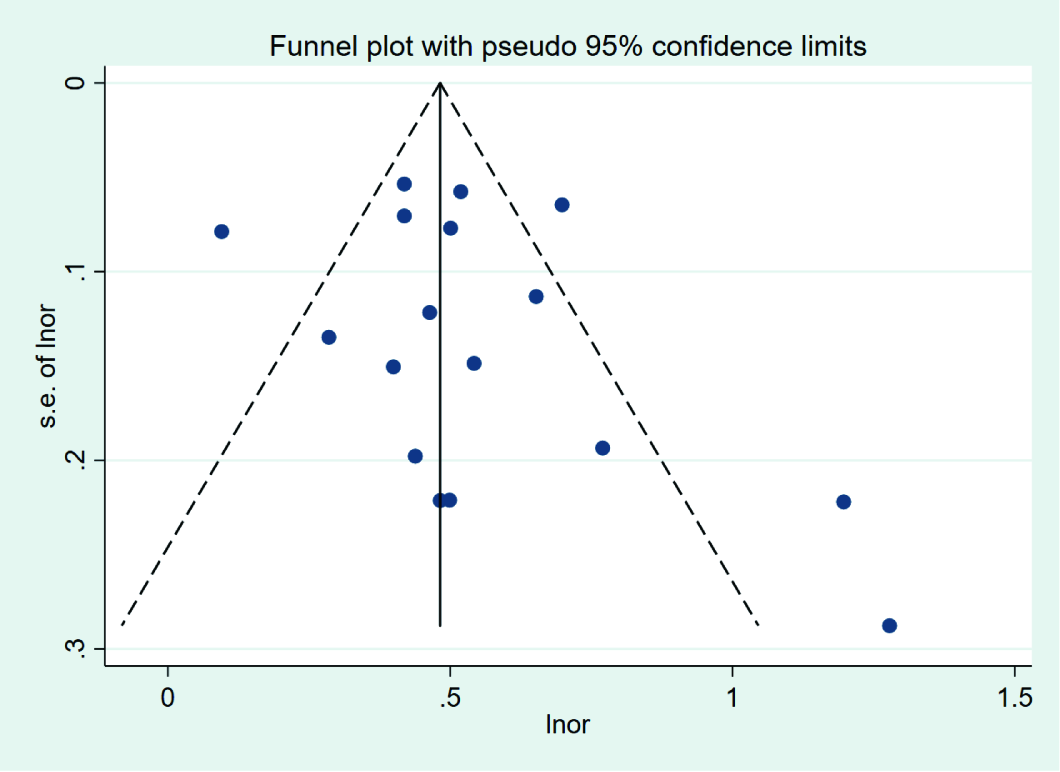

Supplement: Supplementary file 1 [file Table1.docx]
